# Supplementary material for: Autoinhibition and activation of myosin VI revealed by its cryo-EM structure
Source: Nat Commun. 2024 Feb 8;15:1187. doi: 10.1038/s41467-024-45424-7 (PMC10853514; doi:10.1038/s41467-024-45424-7)
Supplement: Supplementary file 1 — Supplementary Information [file 41467_2024_45424_MOESM1_ESM.pdf]

Autoinhibition and activation of myosin VI revealed by its cryo-EM structure

Supplementary Information

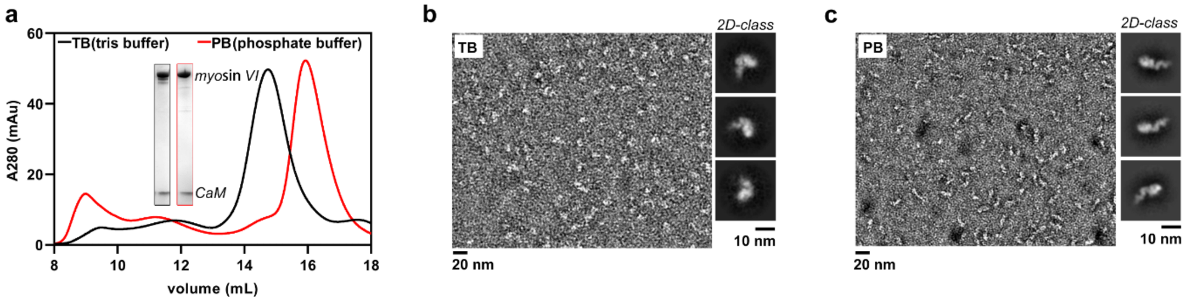

Supplementary Figure 1: Dynamic transition of myosin VI between TB and PB conditions. a, SEC profiles and SDS-PAGE results of myosin VI in the TB and PB conditions. **b, c,** Negative-staining results of myosin VI in TB (**b**) and PB (**c**), respectively, with 2D classification revealing the two different molecular shapes.

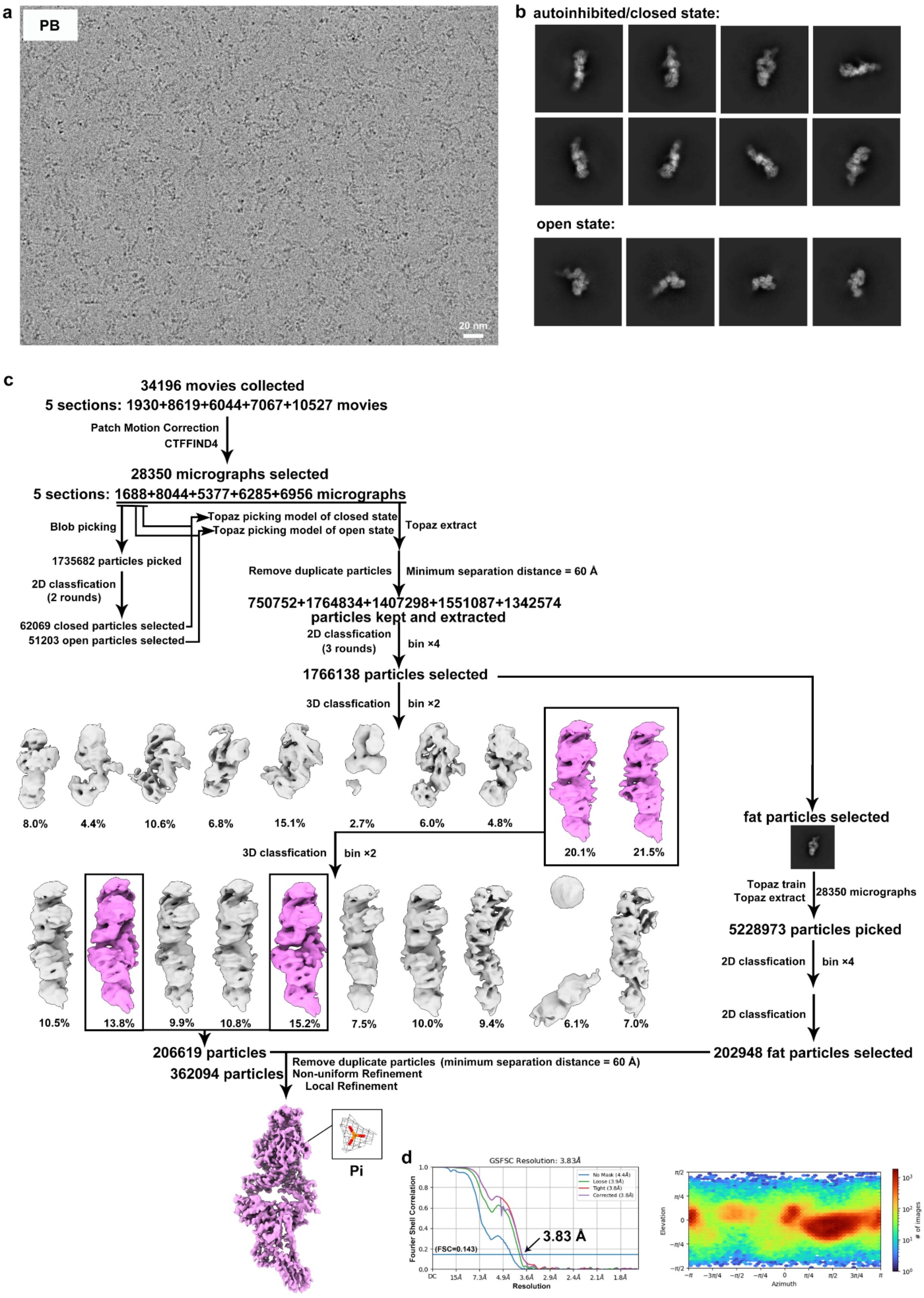
a
PB
20 nm
b autoinhibited/closed state:
open state:
c
34196 movies collected
5 sections: 1930+8619+6044+7067+10527 movies
Patch Motion Correction
CTFFIND4
28350 micrographs selected
5 sections: 1688+8044+5377+6285+6956 micrographs
Blob picking
Topaz picking model of closed state
Topaz picking model of open state
Topaz extract
1735682 particles picked
2D classfication
(2 rounds)
62069 closed particles selected
51203 open particles selected
Remove duplicate particles
Minimum separation distance = 60 Å
750752+1764834+1407298+1551087+1342574
particles kept and extracted
2D classfication
(3 rounds)
bin ×4
1766138 particles selected
3D classfication
bin ×2
8.0%
4.4%
10.6%
6.8%
15.1%
2.7%
6.0%
4.8%
20.1%
21.5%
fat particles selected
3D classfication
bin ×2
Topaz train
Topaz extract
28350 micrographs
5228973 particles picked
2D classfication
bin ×4
2D classfication
10.5%
13.8%
9.9%
10.8%
15.2%
7.5%
10.0%
9.4%
6.1%
7.0%
206619 particles
Remove duplicate particles (minimum separation distance = 60 Å)
202948 fat particles selected
362094 particles
Non-uniform Refinement
Local Refinement
Pi
d
GSFSC Resolution: 3.83Å
No Mask (4.4Å)
Loose (3.9Å)
Tight (3.8Å)
Corrected (3.8Å)
Fourier Shell Correlation
(FSC=0.143)
3.83 Å
Resolution
DC
15Å
7.3Å
4.9Å
3.6Å
2.9Å
2.4Å
2.1Å
1.8Å
Elevation
Azimuth
# of images

Supplementary Figure 2: Cryo-EM data processing of myosin VI in the PB condition. a, Raw image of myosin VI in PB. **b,** 2D classification results showing the particles in the autoinhibited/closed and open states. **c,** Step-by-step data processing procedures lead to the generation of a 3.83-Å map for autoinhibited myosin VI with a phosphate bound at the active site. The contour levels for the final overall map and the local map of Pi molecule are set to 0.1. **d,** Representation of gold-standard FSC (FSC=0.143) used for map resolution determination and distribution of particles orientations.

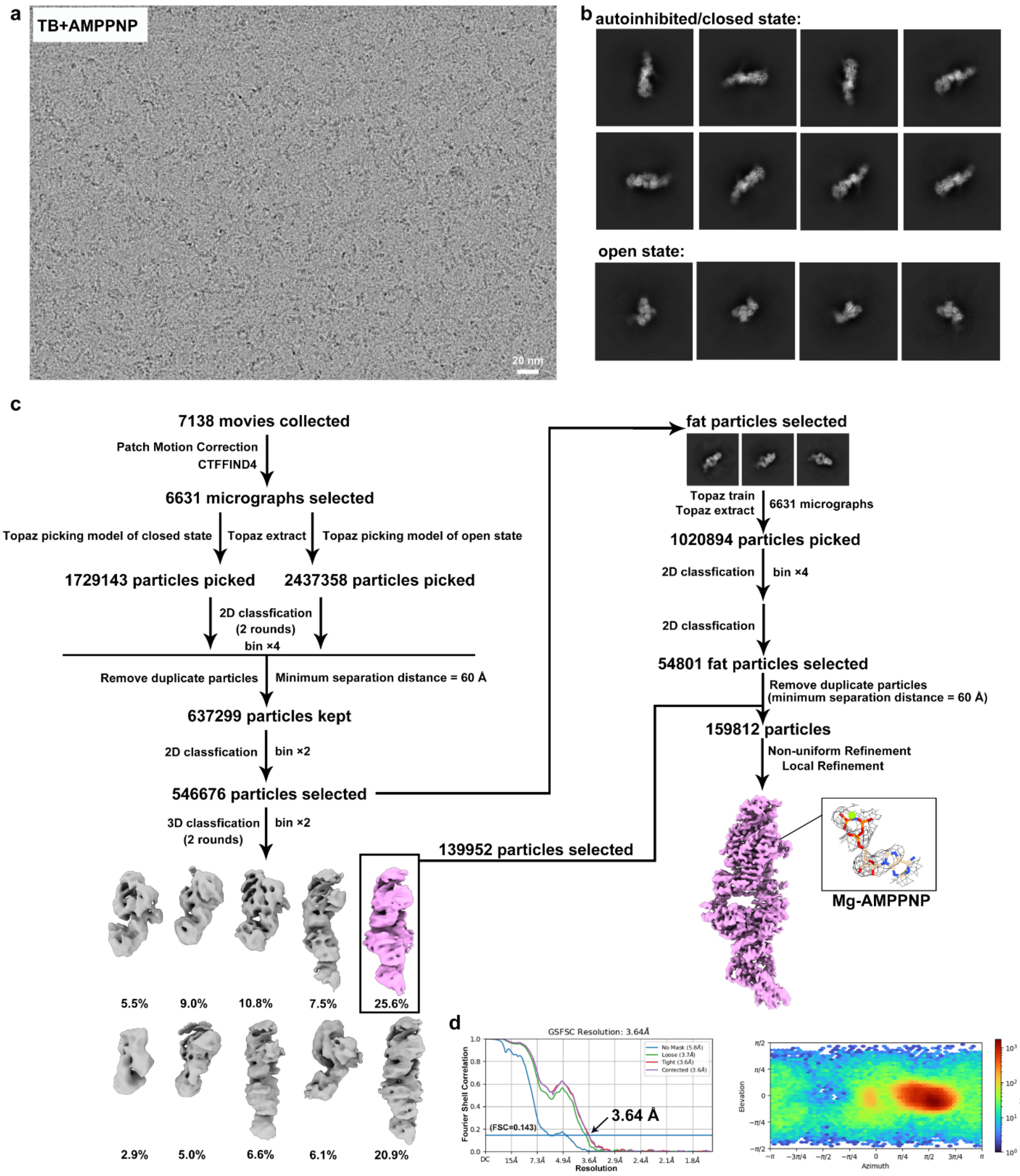

Supplementary Figure 3: Cryo-EM data processing of myosin VI in the TB plus AMPPNP condition. a, Raw image of myosin VI in the condition of TB supplemented with AMPPNP. **b,** 2D classification results showing the particles in the autoinhibited/closed and open states. **c,** Step-by-step data processing procedures lead to the generation of a 3.64-Å map for autoinhibited myosin VI with Mg-AMPPNP bound at the active site. The contour levels for the final overall map and

the local map of AMPPNP molecule are set to 0.1. **d,** Representation of gold-standard FSC (FSC=0.143) used for map resolution determination and distribution of particles orientations.

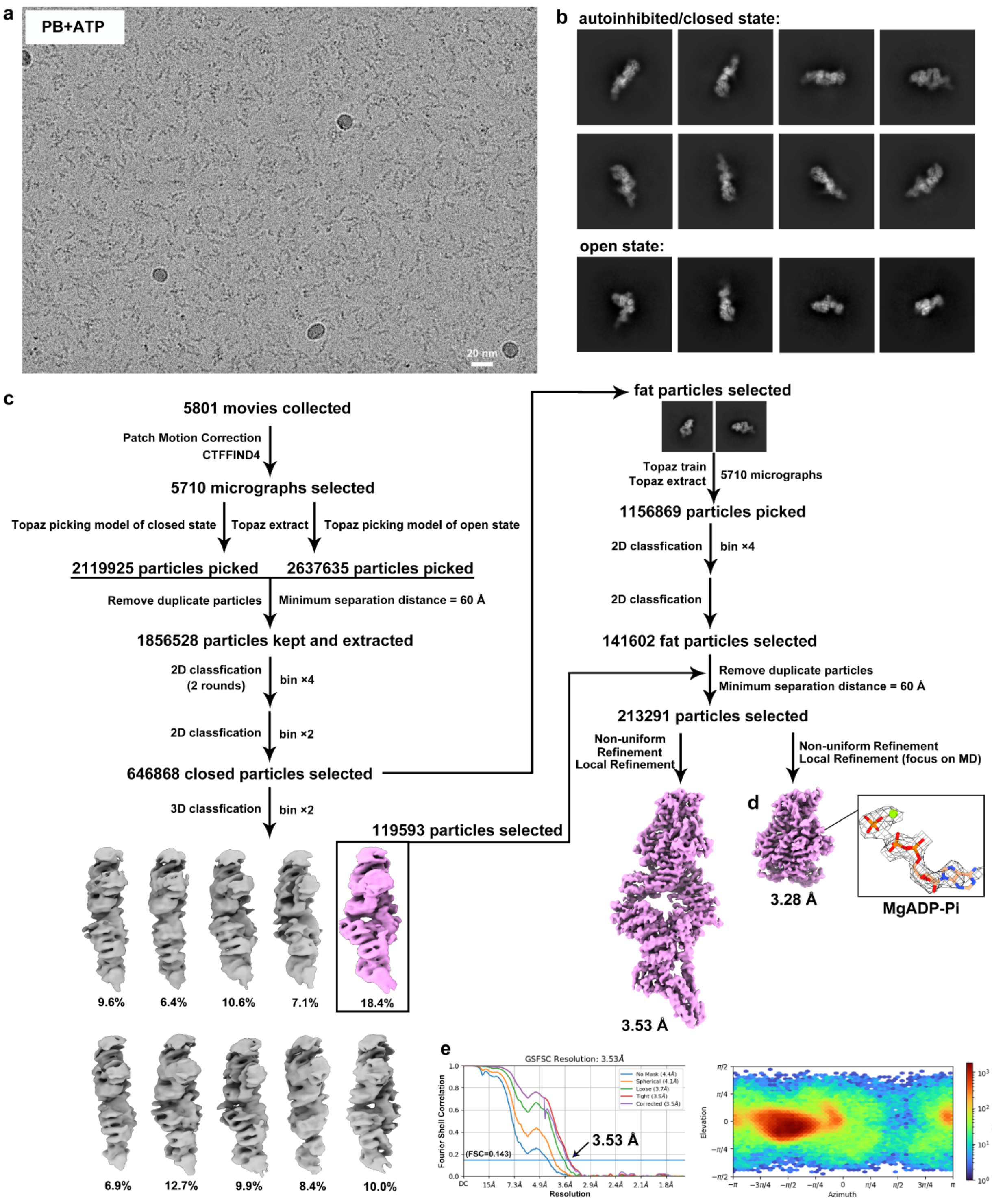

Supplementary Figure 4: Cryo-EM data processing of myosin VI in the PB plus ATP condition. a, Raw image of myosin VI in the condition of PB supplemented with ATP. **b,** 2D classification results showing the particles in the autoinhibited/closed and open states. **c,** Step-by-step data processing procedures lead to the generation of a 3.53-Å map for autoinhibited myosin

VI. The contour level for the final overall map is set to 0.1. **d,** Local refinement focusing on the MD to obtain a 3.28-Å map for confirmation of MgADP-Pi bound at the active site. **e,** Representation of gold-standard FSC (FSC=0.143) used for map resolution determination and distribution of particles orientations.

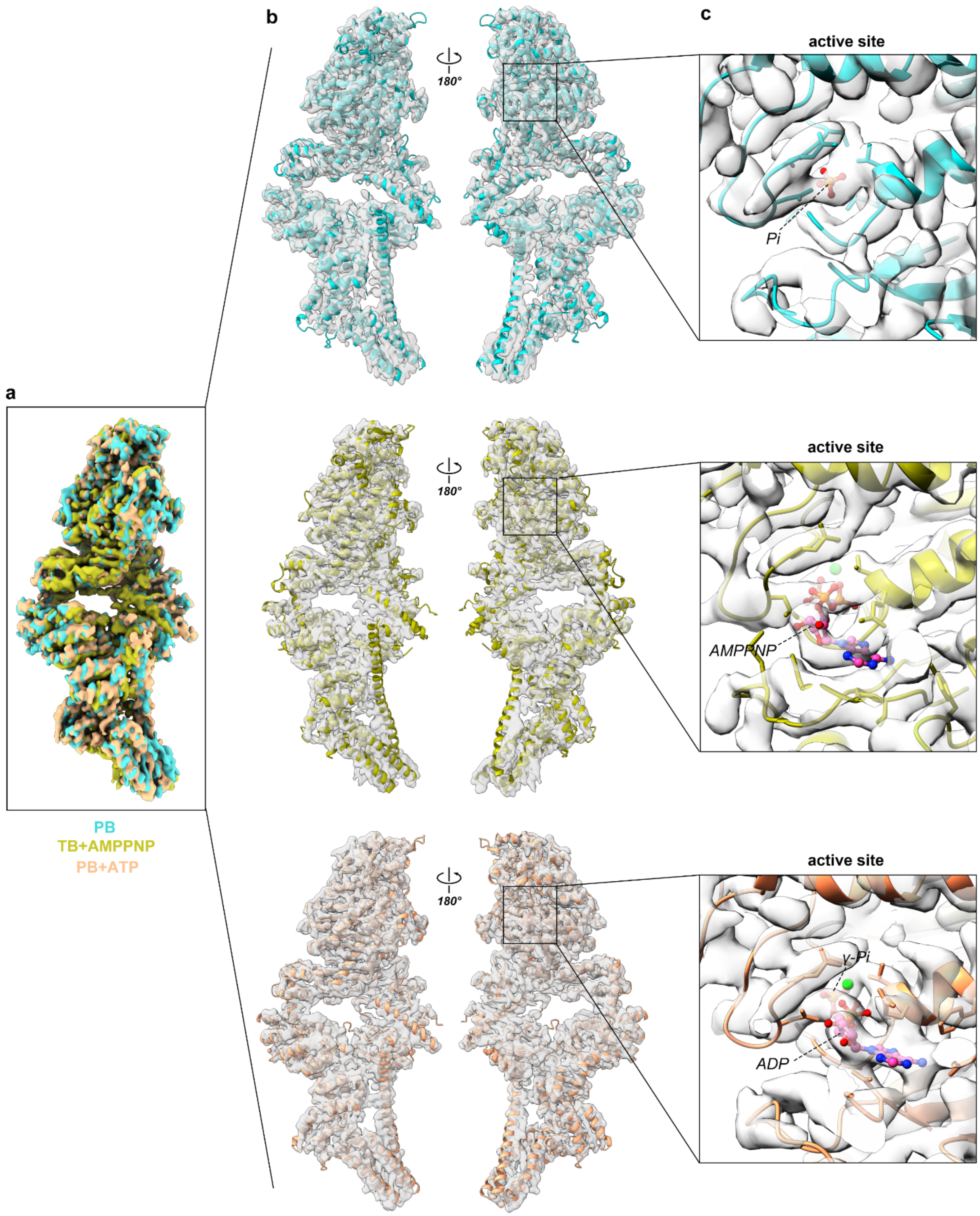

Supplementary Figure 5: Cryo-EM map comparison of myosin VI in the three different conditions of PB, TB plus AMPPNP and PB plus ATP. a, Overlapping of the maps generated from the autoinhibited state of myosin VI in the three different conditions. **b,** Individual maps in a

map-fit-model mode to show the similar overall conformations. **c,** Zoom-in views of the active site in a density-fit-model mode to show the different densities for the bound phosphate and nucleotides. Map contour level = 0.1.

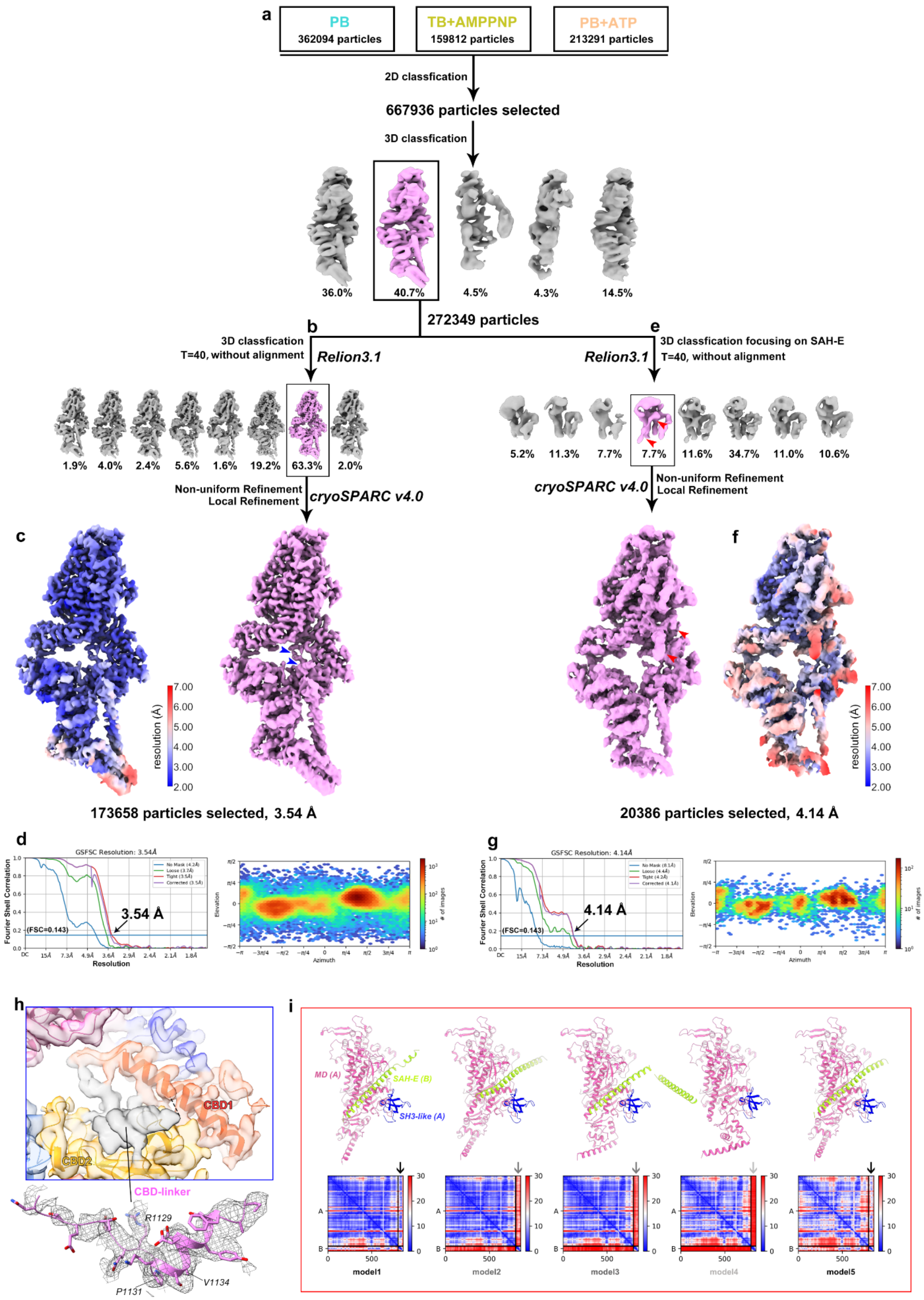
a
PB
362094 particles
TB+AMPPNP
159812 particles
PB+ATP
213291 particles
2D classfication
667936 particles selected
3D classfication
36.0%
40.7%
4.5%
4.3%
14.5%
272349 particles
b
3D classfication
T=40, without alignment
Relion3.1
1.9%
4.0%
2.4%
5.6%
1.6%
19.2%
63.3%
2.0%
Non-uniform Refinement
Local Refinement
cryoSPARC v4.0
e
3D classfication focusing on SAH-E
T=40, without alignment
Relion3.1
5.2%
11.3%
7.7%
7.7%
11.6%
34.7%
11.0%
10.6%
cryoSPARC v4.0
Non-uniform Refinement
Local Refinement
c
f
resolution (Å)
7.00
6.00
5.00
4.00
3.00
2.00
173658 particles selected, 3.54 Å
20386 particles selected, 4.14 Å
d
GSFSC Resolution: 3.54Å
Fourier Shell Correlation
Resolution
(FSC=0.143)
3.54 Å
Elevation
Azimuth
# of images
g
GSFSC Resolution: 4.14Å
(FSC=0.143)
4.14 Å
h
CBD1
CBD2
CBD-linker
R1129
P1131
V1134
i
MD (A)
SAH-E (B)
SH3-like (A)
model1
model2
model3
model4
model5

Supplementary Figure 6: Cryo-EM data processing and structure determination of autoinhibited myosin VI. a-g, Step-by-step procedures of further data processing to combine the autoinhibited particles of myosin VI from the different conditions. Additional 3D classifications using different settings in Relion were performed to generate two final maps for the overall structure determination: one without the SAH-E density (**b, c**) and one with the SAH-E density (indicated by red arrowheads) (**e, f**). The representations of gold-standard FSC (FSC=0.143) to determine the map resolution and the distribution of particles orientation for these two maps are shown in panels **d** and **g**, respectively. The contour levels for the final overall maps are set to 0.1. **h,** Assignment of the CBD-linker based on the interpretation of the cryo-EM density between CBD1 and CBD2. Map contour level = 0.1. **i,** AlphaFold2-powered structure prediction results of the head in complex with the SAH-E. The representations of all five predicted models and the corresponding predicted alignment error (PAE) maps. The model 1 and 5 are used to help the modeling of SAH-E in the autoinhibited myosin VI structure.

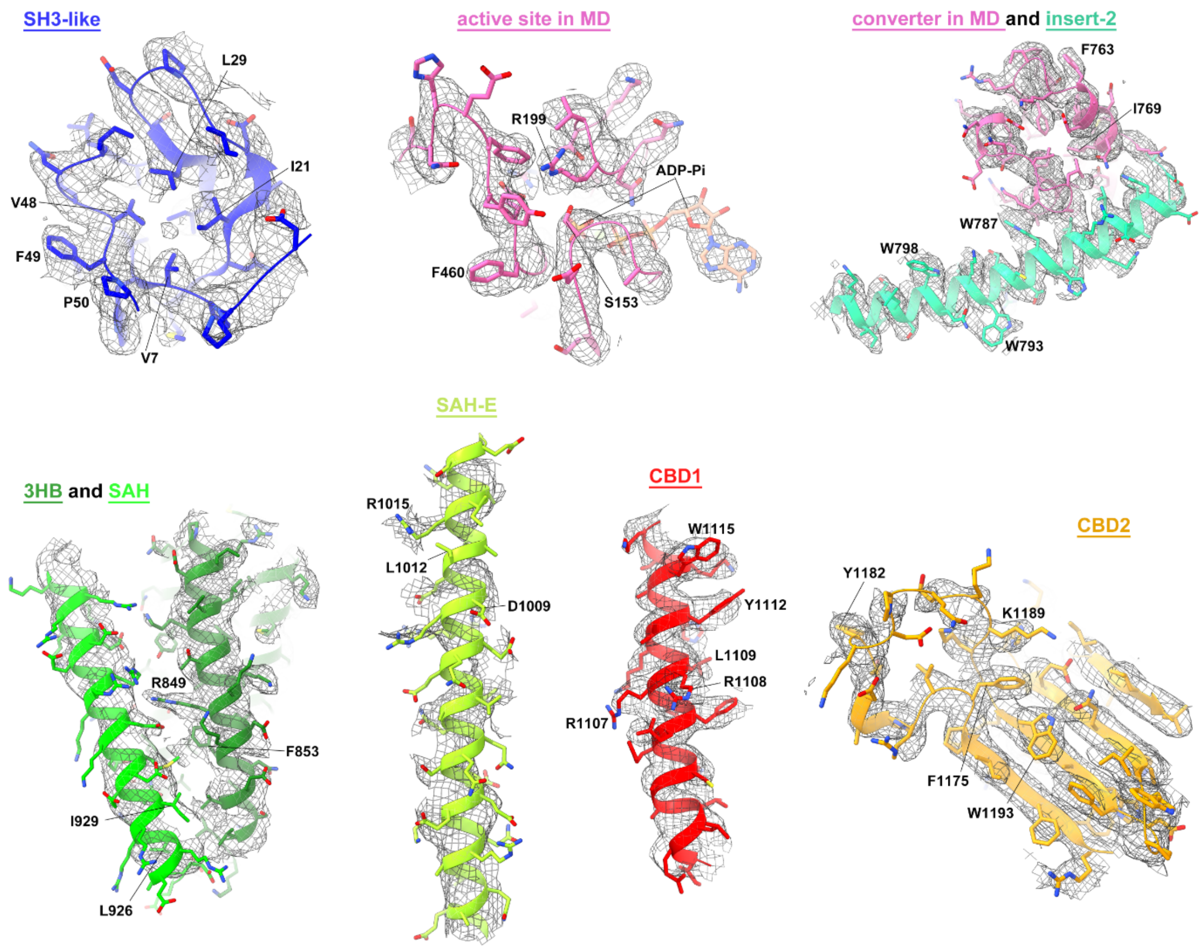

Supplementary Figure 7: Densities of several representative regions in the autoinhibited myosin VI. The density maps are presented with the corresponding structural models overlapped. Map counter lever = 0.1.

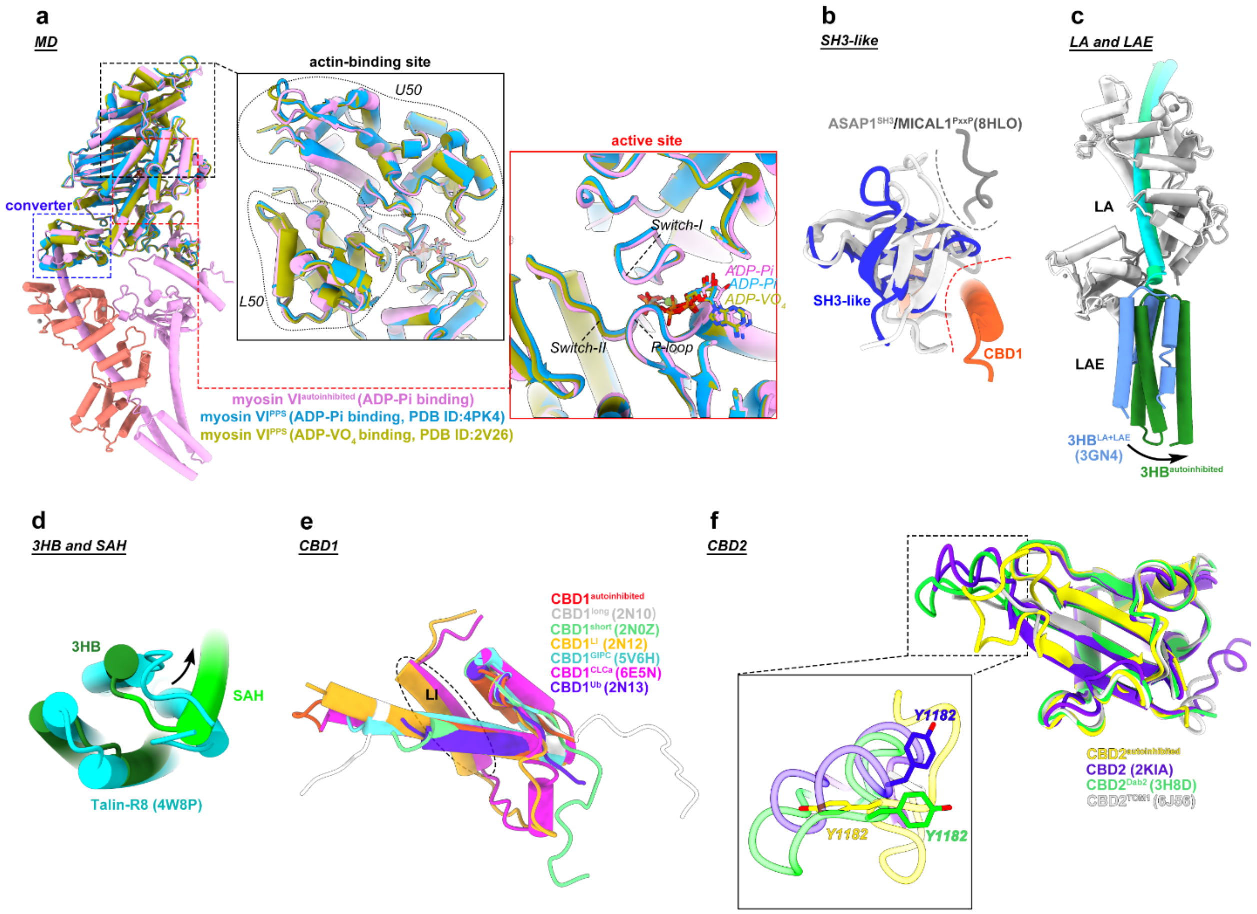

Supplementary Figure 8: Structural comparison of myosin VI in the different conditions. a, Structural comparison of the MDs in the autoinhibited and PPS states to indicate their similarity in the actin-binding site, the active site and the converter. The ADP-Pi-bound MD structure (PDB ID: 4PK4) in the PPS state was determined in a condition with Pi soaking. **b,** The binding of the SH3-like domain to CBD1 in the autoinhibited myosin VI. The CBD1-binding surface on the SH3-like domain is different with the binding pocket in a typical SH3 domain for the recognition of the proline-rich motif. **c,** A rotational change between the isolated structure of the LA and LAE and the LA and LAE region in the autoinhibited structure. **d,** The formation of a loose 4HB between the LAE and SAH in the autoinhibited structure. Compared to the typical 4HB structure of the R8 domain in talin, the packing between the LAE and SAH is much looser, as indicated by a shift of the SAH. **e,** Structural alignment of the CBD1 structures in the indicated conditions. The LI region can be observed in the CLCa-bound condition. **f,** Structural alignment of the CBD2 structures to indicate a conformational change of a loop. The loop is shown with a zoomed-in view to indicate the sidechain orientations of Y1182 in the different CBD2 structures.

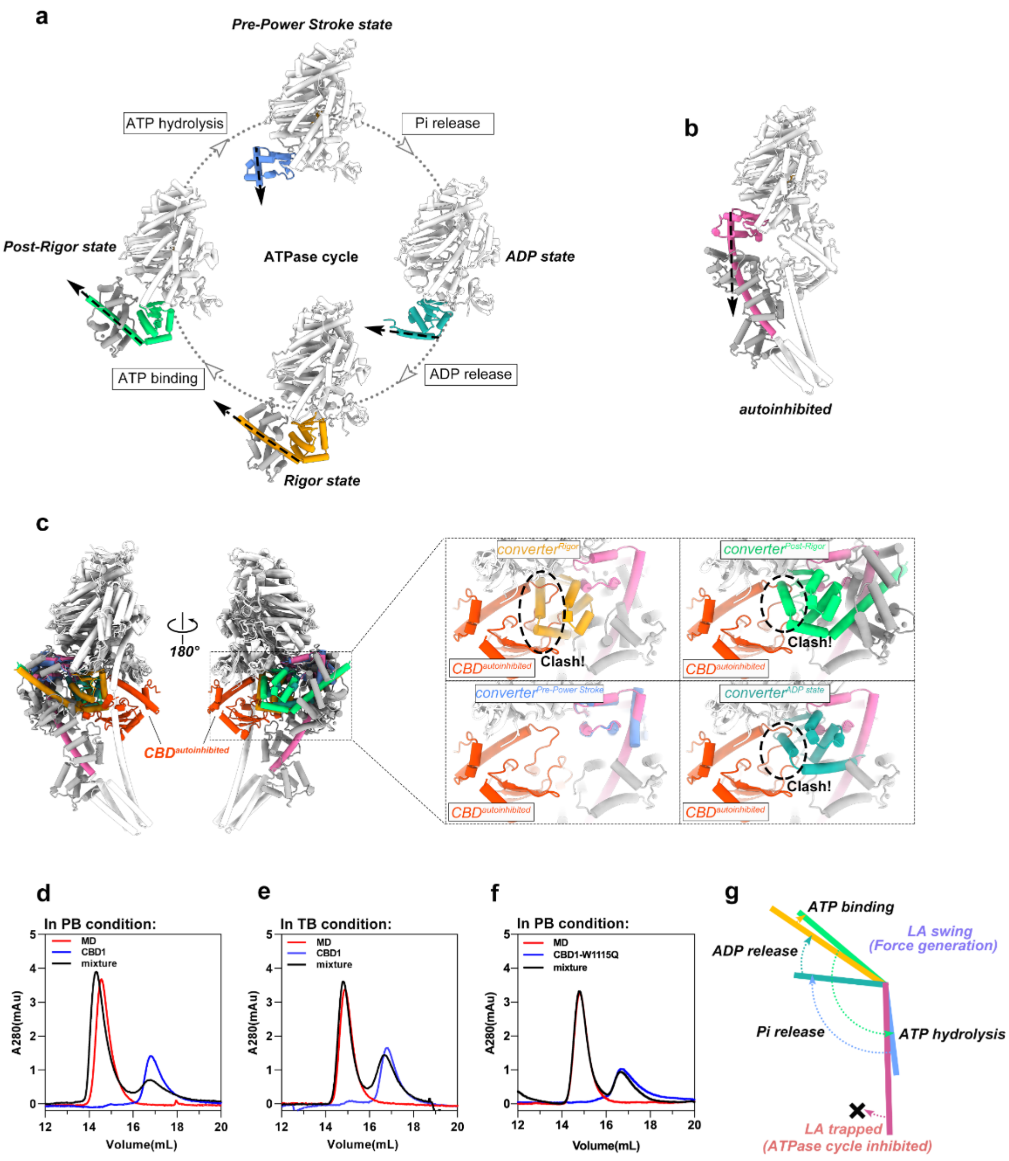

Supplementary Figure 9: Structural and biochemical analyses of the impact of the MD in the different states on myosin VI autoinhibition formation. a, Structural comparison of the MD and LA in the different states of the ATPase cycle, including Pre-Power Stroke state (4PK4), ADP state (6BNW), Rigor state (PDB ID: 2BKI) and Post-Rigor state (2VB6). The different orientations of LA in these states indicate the swing of LA during the ATPase cycle. The extension direction of LA is indicated by black arrows. **b,** The locked orientation of LA in the autoinhibited state of myosin VI. **c,** Structural alignments of the MDs in different states and the autoinhibited myosin VI. Except for the Pre-Power Stroke state, atomic clashes would occur between the CBD in the autoinhibited state and the converter of MD in the Rigor, Post-Rigor, or ADP state. **d-f,** aSEC analyses of the MD/CBD1 interaction. **g,** Schematic comparison showing the swing of LA during

the ATPase cycle. As the swing of the LA is coupled with ATP hydrolysis to generate force, the trapped LA in the autoinhibited state inhibits ATPase activity.

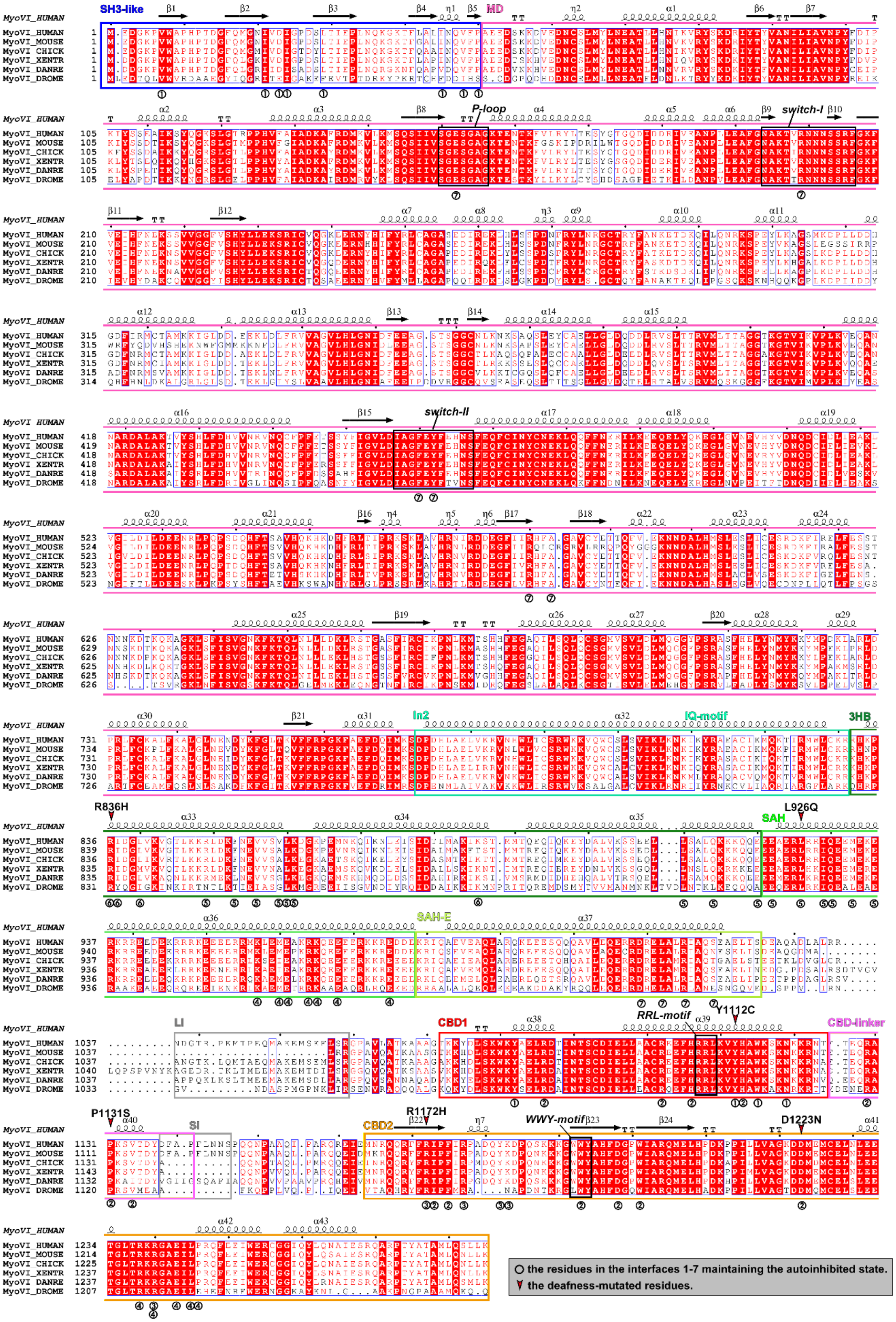

SH3-like
MD
P-loop
switch-I
switch-II
In2
IQ-motif
3HB
R836H
L926Q
SAH
SAH-E
LI
CBD1
RRL-motif
Y1112C
CBD-linker
P1131S
SI
CBD2
R1172H
WWY-motif
D1223N
MyoVI_HUMAN
MyoVI_MOUSE
MyoVI_CHICK
MyoVI_XENTR
MyoVI_DANRE
MyoVI_DROME
the residues in the interfaces 1-7 maintaining the autoinhibited state.
the deafness-mutated residues.

Supplementary Figure 10: Sequence alignment of myosin VI from different species. The domains in myosin VI are boxed and labeled. The P-loop, switch-I and switch-II at the active site and the cargo-binding motifs of RRL and WWY in the CBD region are also indicated. Residues involved in maintaining myosin VI autoinhibition are labeled with a black circle and six deafness-associated mutations located at the interfaces are further highlighted by red arrow heads.

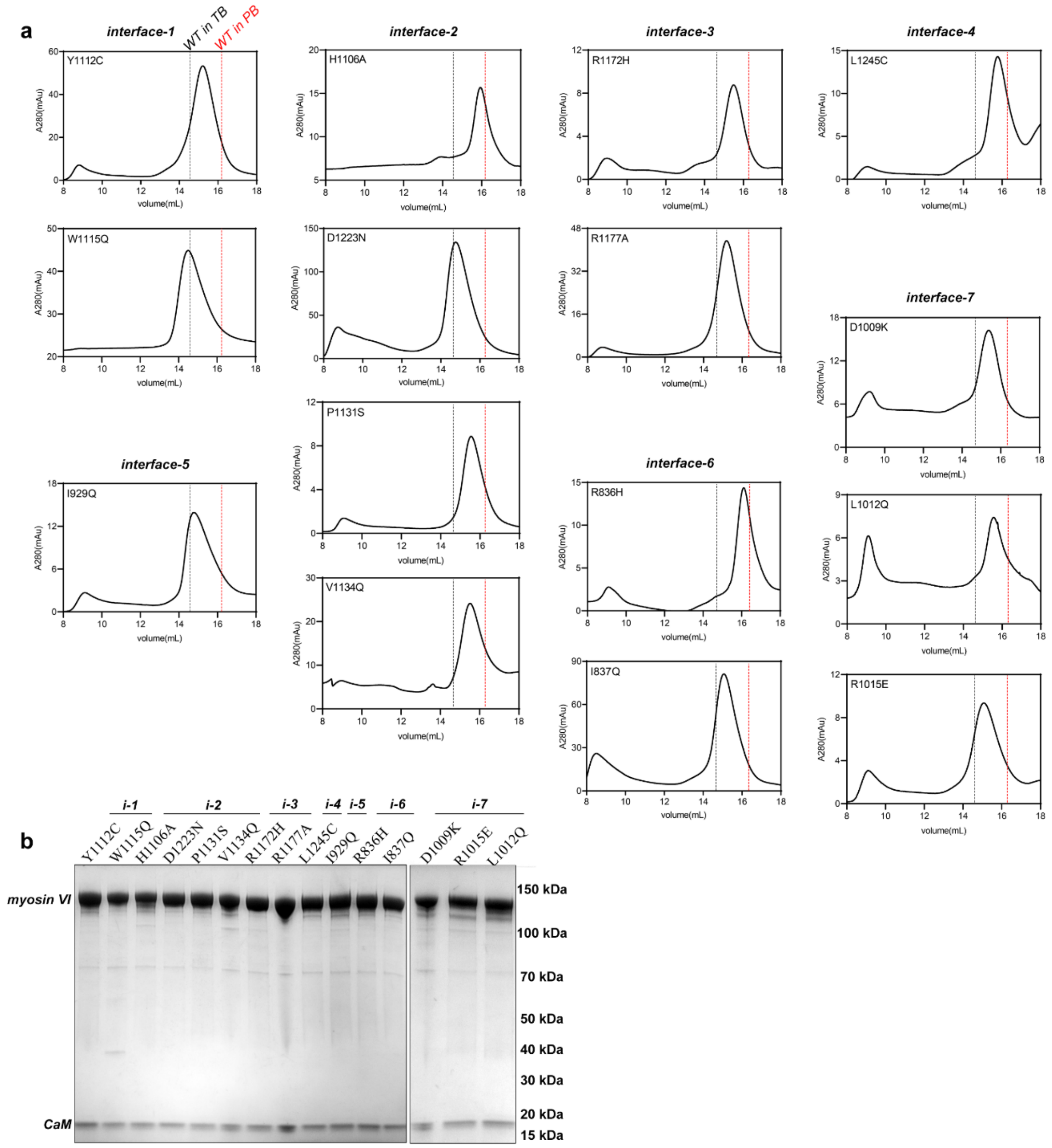

Supplementary Figure 11: Purification of mutated myosin VI protein samples. a, SEC profiles of the samples in the purification. The elution peaks of myosin VI in the conditions of TB and PB, respectively, are indicated for comparison. **b,** SDS-PAGE analysis of the purified protein samples.

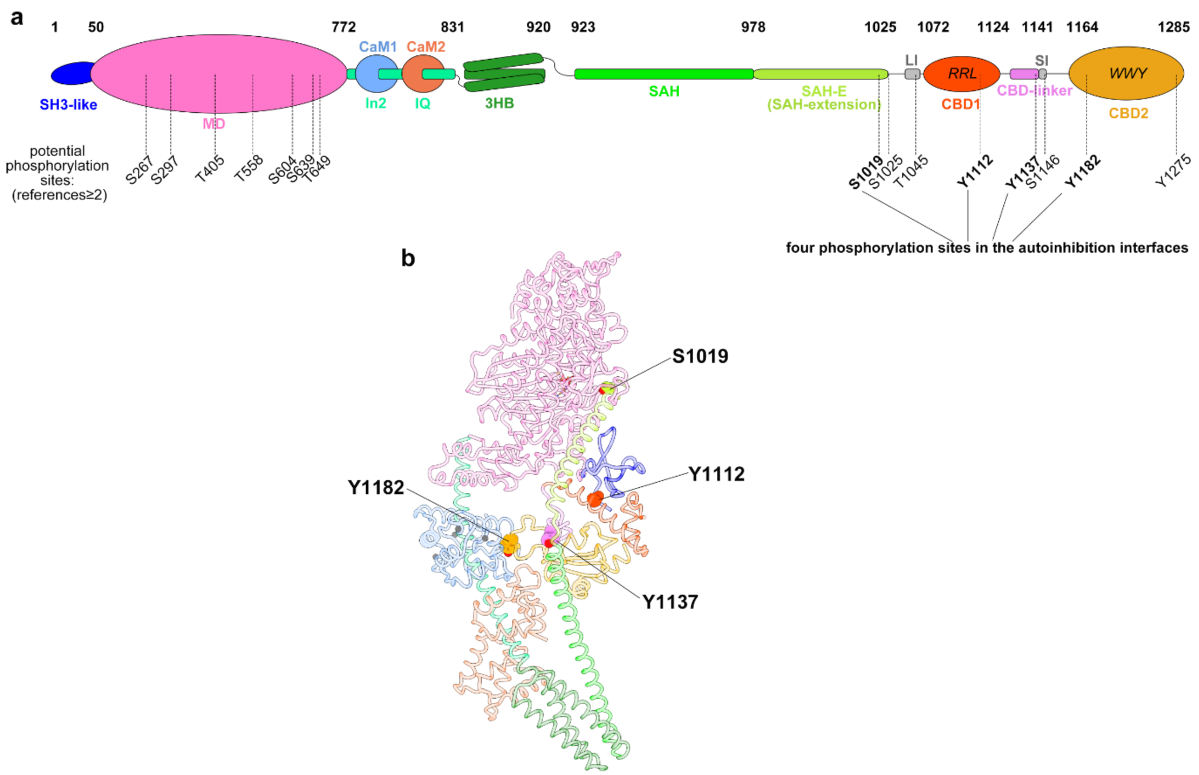

Supplementary Figure 12: Potential activity regulation of myosin VI phosphorylation. a, Identified phosphorylation sites in myosin VI. Phosphorylation sites were obtained from the PhosphoSitePlus database (https://www.phosphosite.org) with a filter of_references⩾2. The phosphorylation sites located in the autoinhibitory interfaces were highlighted in bold. **b,** Four phosphorylation sites located at the interfaces of the autoinhibited myosin VI structure.

Supplementary Table 1: The statistics of cryo-EM data collection, process, model refinement and validation.

Data collection and process	Myosin VI (PB)	Myosin VI (TB+AMPPNP)	Myosin VI (PB+ATP)
Microscope	Titan Krios G3	Titan Krios G3	Titan Krios G3
Detector	Gatan K3	Gatan K3	Gatan K3
Voltage(keV)	300	300	300
Magnification	105000×	105000×	105000×
Total electron exposure (e^- /Å^2)	50	50	50
Exposure time (s)	2.0	2.0	2.0
Electron exposure per frame (e^- /Å^2)	1.56	1.56	1.56
Defocus range (μm)	-1.5 to -2.5	-1.5 to -2.5	-1.5 to -2.5
Pixel size (Å)	0.4135	0.4135	0.4135
Initial particle images (no.)	6816545	1871852	1856528
Final particle images (no.)	173658		20386 (with SAH-E)
Map resolution (Å)	3.54		4.14
FSC threshold	0.143		0.143
Map resolution range (Å)	2.0-6.8		2.4-7.3
Map sharpening B factor (Å^2)	-115.7		-82.0
Symmetry imposed	C1		C1
EMDB code	EMD-37260		EMD-37261
**Refinement**			
Initial model used (PDB code)	2V26, 3GN4, 6OBI, 2N12, 2KIA and AF2-predicted models		
Model composition			
Non-hydrogen atoms	11859		
Protein residues	1455		
Ligands	7		
R.M.S.D. from ideal geometry			
Bond lengths (Å)	0.006		
Bond angles (°)	0.752		
PDB code	8W41		

**Validation**	
CC_mask	0.71
Clashscore	18.39
MolProbity score	2.07
Poor rotamers (%)	0
Ramachandran plot statistics	
Favored (%)	95.63
Allowed (%)	4.37
Disallowed (%)	0
