# Supplementary material for: Autoinhibition and activation of myosin VI revealed by its cryo-EM structure
Source: Nat Commun. 2024 Feb 8;15:1187. doi: 10.1038/s41467-024-45424-7 (PMC10853514; doi:10.1038/s41467-024-45424-7)
Supplement: Supplementary file 3 — Description of additional supplementary files [file 41467_2024_45424_MOESM3_ESM.pdf]

DESCRIPTION FOR ADDITIONAL SUPPLEMENTARY FILES

Supplementary Movie 1: 3.54- Å Cryo-EM map and atomic model of myosin VI in the autoinhibited state.

Supplementary Movie 2: 4.14- Å Cryo-EM map and atomic model of myosin VI with SAHE in the autoinhibited state.
