# Supplementary material for: Autoinhibition and activation of myosin VI revealed by its cryo-EM structure
Source: Nat Commun. 2024 Feb 8;15:1187. doi: 10.1038/s41467-024-45424-7 (PMC10853514; doi:10.1038/s41467-024-45424-7)
Supplement: Supplementary file 6 — Reporting Summary [file 41467_2024_45424_MOESM6_ESM.pdf]

nature portfolio

Corresponding author(s): Zhiyi Wei

Last updated by author(s): Jan 12, 2024

Reporting Summary

Nature Portfolio wishes to improve the reproducibility of the work that we publish. This form provides structure for consistency and transparency in reporting. For further information on Nature Portfolio policies, see our Editorial Policies and the Editorial Policy Checklist.

Statistics

For all statistical analyses, confirm that the following items are present in the figure legend, table legend, main text, or Methods section.

n/a	Confirmed	
☐	☒	The exact sample size (*n*) for each experimental group/condition, given as a discrete number and unit of measurement
☐	☒	A statement on whether measurements were taken from distinct samples or whether the same sample was measured repeatedly
☒	☐	The statistical test(s) used AND whether they are one- or two-sided<br>*Only common tests should be described solely by name; describe more complex techniques in the Methods section.*
☒	☐	A description of all covariates tested
☒	☐	A description of any assumptions or corrections, such as tests of normality and adjustment for multiple comparisons
☐	☒	A full description of the statistical parameters including central tendency (e.g. means) or other basic estimates (e.g. regression coefficient) AND variation (e.g. standard deviation) or associated estimates of uncertainty (e.g. confidence intervals)
☒	☐	For null hypothesis testing, the test statistic (e.g. *F*, *t*, *r*) with confidence intervals, effect sizes, degrees of freedom and *P* value noted<br>*Give P values as exact values whenever suitable.*
☒	☐	For Bayesian analysis, information on the choice of priors and Markov chain Monte Carlo settings
☒	☐	For hierarchical and complex designs, identification of the appropriate level for tests and full reporting of outcomes
☒	☐	Estimates of effect sizes (e.g. Cohen's *d*, Pearson's *r*), indicating how they were calculated

Our web collection on statistics for biologists contains articles on many of the points above.

Software and code

Policy information about availability of computer code

Data collection	Cryo-EM data collection: SerialEM 3.7.
Data analysis	Cryo-EM data process: cryoSPARC v4.0, CTFFIND4, Topaz, Relion3.1, DeepEMhancer.<br>Structure determination and analysis: WinCoot0.9.4.1, Phenix1.18.2, UCSF-Chimera1.16, UCSF ChimeraX1.3.<br>Biochemical data presentation: Prism 8.3

For manuscripts utilizing custom algorithms or software that are central to the research but not yet described in published literature, software must be made available to editors and reviewers. We strongly encourage code deposition in a community repository (e.g. GitHub). See the Nature Portfolio guidelines for submitting code & software for further information.

Data

Policy information about availability of data

All manuscripts must include a data availability statement. This statement should provide the following information, where applicable:

- Accession codes, unique identifiers, or web links for publicly available datasets
- A description of any restrictions on data availability
- For clinical datasets or third party data, please ensure that the statement adheres to our policy

The cryo-EM density maps of autoinhibited MyoVI have been deposited into EMDB with accession codes EMD-37260 and EMD-37261. The corresponding atomic model has been also deposited in PDB with accession code 8W41.

Research involving human participants, their data, or biological material

Policy information about studies with human participants or human data. See also policy information about sex, gender (identity/presentation), and sexual orientation and race, ethnicity and racism.

Reporting on sex and gender	n/a
Reporting on race, ethnicity, or other socially relevant groupings	n/a
Population characteristics	n/a
Recruitment	n/a
Ethics oversight	n/a

Note that full information on the approval of the study protocol must also be provided in the manuscript.

Field-specific reporting

Please select the one below that is the best fit for your research. If you are not sure, read the appropriate sections before making your selection.

☒ Life sciences ☐ Behavioural & social sciences ☐ Ecological, evolutionary & environmental sciences

For a reference copy of the document with all sections, see nature.com/documents/nr-reporting-summary-flat.pdf

Life sciences study design

All studies must disclose on these points even when the disclosure is negative.

Sample size	No any statistical methods were used to predetermine sample size.<br>For cryo-EM structure determination, 34196 movies of myosin VI in the PB condition, 7138 movies of myosin VI in the TB+AMPPNP condition and 5801 movies of myosin VI in the PB+ATP condition were collected.<br>In vitro ATPase assay was performed with three repeats.
Data exclusions	No data exclusion was pre-established.<br>Junk micrographs in cryo-EM data were manually removed, which is generally accepted in cryo-EM data processing since single particle analysis in cryo-EM is essentially to optimize the best particles for high-resolution map generation.
Replication	Replication is part of single particle analysis in cryo-EM data process. All the cryo-EM structures were determined by two independent half datasets, which was used to determine the map resolution.<br>In vitro ATPase assay was performed with three repeats and all attempts at replication were successful.
Randomization	For cryo-EM data processing, the datasets were randomly split into two halves for structure determination. Randomization is unnecessary and not applicable for the the in vitro biochemical assays in this study.
Blinding	Blinding is unnecessary and not applicable for the in vitro biochemical assays and cryo-EM structure determination in this study.

Reporting for specific materials, systems and methods

We require information from authors about some types of materials, experimental systems and methods used in many studies. Here, indicate whether each material, system or method listed is relevant to your study. If you are not sure if a list item applies to your research, read the appropriate section before selecting a response.

Materials & experimental systems

n/a	Involved in the study	
☒	☐	Antibodies
☐	☒	Eukaryotic cell lines
☒	☐	Palaeontology and archaeology
☒	☐	Animals and other organisms
☒	☐	Clinical data
☒	☐	Dual use research of concern
☒	☐	Plants

Methods

n/a	Involved in the study	
☒	☐	ChIP-seq
☒	☐	Flow cytometry
☒	☐	MRI-based neuroimaging

Eukaryotic cell lines

Policy information about cell lines and Sex and Gender in Research

Cell line source(s)	HEK 293F cells (Thermo Fisher Scientific) for protein expression.
Authentication	No authentication was performed.
Mycoplasma contamination	No mycoplasma contamination was found under observation by microscope.
Commonly misidentified lines (See ICLAC register)	n/a
